# Phenotypic antimicrobial resistance in *Escherichia coli* strains isolated from swine husbandries in North Western Germany – temporal patterns in samples from laboratory practice from 2006 to 2017

**DOI:** 10.1186/s12917-020-2268-z

**Published:** 2020-02-03

**Authors:** C. Moennighoff, N. Thomas, F. Nienhaus, M. Hartmann, A. Menrath, J. Merkel, H. Detlefsen, L. Kreienbrock, I. Hennig-Pauka

**Affiliations:** 1 Field Station for Epidemiology in Bakum, University of Veterinary Medicine Hannover, Hanover, Germany; 2 Department of Biometry, Epidemiology and Information Processing, WHO Collaborating Centre for Research and Training for Health at the Human-Animal-Environment Interface, University of Veterinary Medicine Hannover, Hanover, Germany; 3 Department of Infectious Diseases, Institute for Microbiology, University of Veterinary Medicine Hannover, Hanover, Germany

**Keywords:** Routine data, Minimal inhibitory concentration, Pig production, Epidemiology

## Abstract

**Background:**

Since 2011, antibiotic usage has decreased continuously in livestock in Germany. Whether this is accompanied by a reduction in bacterial antimicrobial resistance has not been proven so far. In this study 3054 *Escherichia coli (E. coli)* isolates from pigs which had suffered from disease on 2161 farms in North Western Germany were evaluated retrospectively from 2006 to 2017 for trends in their antimicrobial resistance pattern. Data were substantially related to the “pre-reduction period” and were therefore suggested as a basis for this task.

Minimal inhibitory concentrations for selected antimicrobial substances were evaluated for *E. coli* strains isolated from different organs of diseased swine sampled for routine diagnostic. In total, 81% of *E. coli* were isolated from faeces or the gastrointestinal tract, 11% from the genito-urinary tract and 8% from other organs. Susceptibility testing and classification of isolates in accordance with clinical cut-offs followed the Clinical and Laboratory Standards Institute (CLSI). If no clinical cut-offs were available for the respective combination of species, substance and organ, other published clinical cut-offs were used.

**Results:**

Differences in susceptibility patterns between isolates from the gastrointestinal and genito-urinary tract were found for most substances. Isolates from the genito-urinary tract were less frequently resistant to ampicillin, apramycin, colistin, neomycin, spectinomycin and tetracycline and more frequently resistant to enrofloxacin and florfenicol.

A multifactorial logistic regression model revealed time-dependent decreases in frequency of resistant isolates for neomycin, spectinomycin and tetracycline. For colistin, the highest percentage of resistant isolates with 16.0% was found in 2015 followed by a decrease to the level of 2009–2010 in 2017. A decrease in frequencies of ampicillin-resistant isolates was dependent on the age-group and time period. Irrespective of the year, less than 15% *E. coli* isolates were resistant to apramycin, cephalosporins, colistin, enrofloxacin, florfenicol, gentamicin and neomycin.

**Conclusion:**

An overall time-dependent decrease in the percentage of resistant *E. coli* isolates was found for some substances. These data from diseased animals indicate an impact of a general reduction in antibiotic usage on development of bacterial antimicrobial resistance in the field and can support the decision-making of swine practitioners for treatment options in swine.

## Background

The development of antimicrobial resistance (AMR) is a natural phenomenon in bacteria. Nonetheless, antibiotic treatment either in the recommended dosage, under- or overdosed is triggering the global expansion of AMR due to shifts towards more resistant bacterial populations [1, 2]. In the human health care sector, a close link between the frequency of antibiotic usage and the prevalence of resistant bacteria exists [3, 4]. This association was also shown for livestock animals, which has a high impact on consumer protection due to the cross-linking of animal and human bacterial reservoirs [5]. Epidemiological studies revealed either an increase in antimicrobial resistance in populations treated more often with antibiotics [6, 7], or a beneficial effect on resistance levels after the introduction of national antimicrobial usage (AMU) reduction campaigns [8]. Under defined experimental conditions, the effect of a selective pressure by antimicrobial usage on the frequency of phenotypically resistant bacteria in swine [9] or the total resistome of bacterial populations [10] was proven. In general, under field conditions, in livestock, the association between AMU and AMR is difficult to assess because additional factors such as vaccination, feed supplements, hygiene conditions and management characteristics also have an impact [11]. In a European study, farm-specific faecal antimicrobial resistomes defined in a metagenomic approach revealed a clear association between usage of tetracyclines and macrolides with the respective resistance genes, while for other widely used substances, no associations were found [12]. In a longitudinal study on one pig farm, the elimination of *E. coli* carrying the mobile *mcr-1* gene coding for colistin resistance was achieved within a 20-month period after stopping colistin treatment [13].

In order to monitor the development of AMR continuously, several surveillance programmes have been implemented in Europe. In particular, both, the transnationally acting Global Antimicrobial Resistance Surveillance System (GLASS) and the European Antimicrobial Resistance Surveillance System (EARSS) as well as national programmes in the different countries record antimicrobial resistance in bacteria as well as the amount of antibiotics used in animals at different levels, i.e. animals, food and humans.

The collection of farm-level AMU data in different European countries revealed country-specific differences in reported variables, so that data are difficult to compare. In a recent study in nine European countries, overall, AMU was highest in weaners, the majority being administered in feed or water, the most frequent specific treatment indications being intestinal and respiratory disorders [14]. Indications and used antimicrobial substances varied between countries and different age groups [15, 16]. In Germany, data of antimicrobial consumption and resistance in bacteria have been systematically available since 2008 [17]. These data derive from passive surveillance reporting information from 2008, 2010, 2012 and 2015. Since 2011, pharmaceutical companies have had to report the quantities of antibiotics dispensed per year in accordance with the German Pharmaceuticals Act [18] and the DIMDI-Ordinance [19] on Medicinal Products. In 2011 1706 tons of antimicrobial agents were delivered. The most widely given active ingredients were tetracyclines (564 tons), aminopenicillins (528 tons), sulfonamides (185 tons) and macrolides (173 tons). The indication” respiratory disease” was recorded most frequently in weaners and sows and mainly treated with amoxicillins and tetracyclines [20]. In addition, in sows, reproductive and fundamental disorders were main causes for antibiotic treatment. Reproductive disorders were mainly treated with tetracyclines, followed by amoxicillin [20]. In comparison with 2011, the amount of antimicrobials sold more than halved in 2017 [16]. Also, with regard to treatment frequency in 2011, 2013 and 2014, a clear reduction was observed in German pig holdings [21, 22]. In the years 2013–2015, fatteners were mainly treated with tetracyclines and penicillines, accounting for 60% of antibiotic treatments, while in weaners, amoxicillin and colistin were most often used [22]. In suckling piglets, treatments with macrolides and penicillines were most frequent [22]. Obligatory monitoring of the usage of antibiotic substances in fattening pigs and weaners, as well as in poultry and beef cattle was enforced in the 16th Amendment of the German Pharmaceuticals Act in 2014 [18]. Since then, antibiotic usage data of swine have been collected in a central database and the treatment frequency for the respective farm is calculated, being compared with the countries’ median and sanctioned if exceeding the upper quartile value [23].

Due to the general nature of these data, so far, linking AMU to frequencies of AMR is not possible [24], and much more specialised livestock information is needed [25]. For assessing antibacterial resistance data from the field, the various resistance mechanisms in different bacterial species must be taken into account [26–28].

The Field Station for Epidemiology of the University of Veterinary Medicine Hannover, Germany is a diagnostic institute located in a swine-dense region in the North Western part of Germany. Since 2006, data of AMR resistance in swine pathogens have been recorded in an institutional database. By using descriptive and inductive statistical methods 3054 data for *E. coli* originating from diseased pigs were evaluated in this study at yearly intervals. The hypothesis behind evaluating routine data was that pronounced changes in AMR over the years in parallel to an observed reduction in AMU would become detectable in a field population.

## Results

### Distribution of antimicrobial resistance

The *E. coli* isolates originated from diseased pigs from 2161 farms in a period from 2006 to 2017.

In total, frequencies of resistant isolates below 6% were found for cefquinome, ceftiofur, gentamicin, apramycin, florfenicol and enrofloxacin. Highest frequencies above 70% of resistant isolates were found for ampicillin and tetracycline as shown in Table 1. While more than 96% of the isolates were susceptible to third- and fourth-generation cephalosporins, i.e. ceftiofur and cefquinome, this was only the case for 57% of the isolates with respect to the first-generation cephalosporin, cephalothin, with a high percentage of intermediate isolates, namely 33%.

**Table 1:** MIC distribution of *E.coli* isolates from 2006 to 2017 and frequency of resistant isolates

Agent	n	Percentage of isolates with MIC-values (mg/l) of…																		MIC_50_	MIC_90_	Clinical cut-off			Epidemiological cut-off		
																						S	I	R	S	I	R
		0.016	0.026	0.031	0.053	0.063	0.105	0.125	0.25	0.5	1	2	4	8	16	32	64	128	256			%	%	%	%	%	%
Ampicillin	3054	.	.	.	.	.	.	.	.	0.1	1.7	12.8	9.8	1.2	0.3	0.5	73.6	.	.	64.00	64.00	25.61	0.26	74.13	25.61	.	74.39
Apramycin	2969	.	.	.	.	.	.	.	.	.	.	.	93.3	.	1.8	0.5	4.4	.	.	4.00	4.00	95.05	.	4.95	.	.	.
Cefquinome	2969	.	.	.	.	.	.	.	.	96.0	.	0.3	0.4	0.7	2.6	.	.	.	.	0.50	0.50	96.36	0.40	3.23	.	.	.
Ceftiofur	3054	.	.	.	.	.	.	.	2.0	93.6	.	0.4	0.3	0.2	3.4	.	.	.	.	0.50	0.50	96.07	0.26	3.67	95.65	.	4.35
Cephalothin	3054	.	.	.	.	.	.	.	.	.	.	10.2	0.2	46.5	32.9	5.0	5.1	.	.	8.00	32.00	56.91	32.94	10.15	94.89	.	5.11
Colistin	3053	.	.	.	.	.	.	.	81.2	.	7.1	1.1	3.2	7.5	.	.	.	.	.	0.25	4.00	81.17	8.16	10.68	89.32	.	10.68
Enrofloxacin	3054	2.1	.	78.7	.	0.2	.	2.0	5.6	7.2	1.2	0.7	2.40	.	.	.	.	.	.	0.03	0.5	88.51	8.48	3.01	82.91	.	17.09
Florfenicol	3054	.	.	.	.	.	.	.	.	0.8	.	7.1	54.5	32.4	5.2	.	.	.	.	4.00	8.00	62.41	32.42	5.17	100.00	.	.
Gentamicin	3054	.	.	.	.	.	.	0.3	.	1.7	90.3	.	1.7	1.6	2.3	2.1	.	.	.	1.00	1.00	93.98	1.64	4.39	92.31	.	7.69
Neomycin	2969	.	.	.	.	.	.	.	.	.	.	.	84.2	.	1.4	4.7	9.7	.	.	4.00	32.00	84.20	1.38	14.42	84.20	.	15.80
Spectinomycin	3054	.	.	.	.	.	.	.	.	.	.	.	1.7	.	34.4	21.5	12.0	10.3	20.0	32.00	256.00	57.63	12.05	30.32	69.68	.	30.32
Tetracycline	3054	.	.	.	.	.	.	.	0.1	3.8	1.4	15.1	1.6	0.4	2.1	75.7	.	.	.	32.00	32.00	21.87	0.39	77.73	22.27	.	77.73

The distribution of MICs of respective *E. coli* isolates is depicted in Table 1. The MICs for most antimicrobial agents were unimodally distributed. MICs for apramycin, cefquinome, ceftiofur, colistin, enrofloxacin, gentamicin and neomycin showed a unimodal left-skewed distribution, indicating low MICs of mainly susceptible isolates. Interquartile ranges of MICs in the different years were narrow for substances with high susceptibility (e.g. ceftiofur/cefquinome 0.5–0.5 mg/L, gentamicin 1–1 mg/L, neomycin/apramycin 4–4 mg/L, enrofloxacin 0.03–0.03 mg/L, colistin 0.25–0.25 mg/L) or with a high resistance rate in *E. coli* (e.g. tetracycline 32–32 mg/L). Wider interquartile ranges in MICs were found for ampicillin (8–64 mg/L) and spectinomycin (16–128 mg/L). Spectinomycin MICs were bimodally distributed with more than 30% of the isolates having MICs of 16 or lower, while 20% of the isolates were characterised by an MIC of 256. Cephalothin, florfenicol and spectinomycin could not be classified according to their MIC distribution and many isolates were classified as intermediate.

Assessment of susceptibility of isolates according to epidemiological cut-offs resulted in slightly different frequencies of resistant isolates, which were in almost all cases higher or equal to those assessed by clinical cut-offs (Table 1).

### Comparison of isolates from different sampling sites and age-groups

The sampling site as well as age-group were significant factors influencing the relative frequencies of resistant isolates. For that reason, single comparisons (chi-square test) as well as simple and multifactorial logistic regression models were used to elucidate the impact of the factors time period, age-group and sampling site on the frequencies of resistant isolates. Absolute and relative frequencies of isolates from different sampling sites are shown in Table 10. While the percentage of age-groups within the data subset of 956 animals did not change over time, the percentage of samples from the gastrointestinal and the genito-urinary tract (*n* = 2809) differed in the observed time periods with approximately 14% of samples from the genito-urinary tract in 2006–2011 and only 8% from the period 2012–2017 (*p* < 0.0001) (data not shown). Lower frequencies of resistant isolates were found in the genito-urinary tract for ampicillin, apramycin, colistin, neomycin, spectinomycin and tetracycline. In contrast to that, higher frequencies of resistant isolates in the genito-urinary in comparison to the gastrointestinal tract were found for enrofloxacin and florfenicol (Tables 2, 3, 4, and 5, Additional file 1f-g).

**Table 2:** Neomycin resistance

		Resistant isolates		Susceptible isolates		One-factorial log. Reg.		Multi-factorial log. Reg.	
		n	%	n	%	OR	p	OR	p
Time period	2006–2011 (Ref.)	280	15.77	1496	84.23	1	–	1	–
	2012–2017	148	12.85	1004	87.15	0.79	0.029	0.60	0.011
Age-group	Sow (Ref.)	0	0	35	100	1	–	1	–
	Piglet	17	12.98	114	87.02	–	0.949	–	0.951
	Nursery	100	14.66	582	85.34	–	0.949	–	0.950
	Fattening	4	9.09	40	90.91	–	0.949	–	0.953
Sampling site	GIT (Ref.)	363	15.35	2002	84.65	1	–	1	–
	GUT	25	7.69	300	92.31	0.46	0.0003	–	0.958
	others	40	16.81	198	83.19	1.11	0.554	–	0.857

**Table 3:** Spectinomycin resistance

		Resistant isolates		Susceptible isolates		One-factorial log. Reg.		Multi-factorial log. Reg.	
		n	%	n	%	OR	p	OR	p
Time period	2006–2011 (Ref.)	618	39.09	963	60.91	1	–	1	–
	2012–2017	308	27.87	797	72.13	0.60	< 0.0001	0.62	0.003
Age-group	Sow (Ref.)	8	22.22	28	77.78	1	–	1	–
	Piglet	39	312	86	68.8	1.59	0.300	1.93	0.422
	Nursery	206	32.09	436	67.91	1.65	0.220	1.76	0.489
	Fattening	13	31.71	28	68.29	1.62	0.354	1.76	0.512
Sampling site	GIT (Ref.)	783	36.23	1378	63.77	1	–	1	–
	GUT	79	25.73	228	74.27	0.61	0.0003	1.220	0.8104
	others	64	29.36	154	70.64	0.73	0.044	0.685	0.1871

**Table 4:** Tetracycline resistance

		Resistant isolates		Susceptible isolates		One-factorial log. Reg.		Multi-factorial log. Reg.	
		n	%	n	%	OR	p	OR	p
Time period	2006–2011 (Ref.)	1492	82.98	306	17.02	1	–	1	–
	2012–2017	882	70.9	362	29.10	0.5	< 0.0001	0.5	0.0002
Age-group	Sow (Ref.)	25	65.79	13	34.21	1	–	1	–
	Piglet	97	69.78	42	30.22	1.20	0.637	1.00	0.999
	Nursery	557	76.72	169	23.28	1.71	0.127	1.36	0.637
	Fattening	32	64	18	36	0.93	0.862	0.73	0.644
Sampling site	GIT (Ref.)	1967	79.86	496	20.14	1	–	1	–
	GUT	226	67.66	108	32.34	0.53	< 0.0001	0.84	0.874
	others	181	73.88	64	26.12	0.71	0.028	0.96	0.796

**Table 5:** Ampicillin resistance

		Resistant isolates		Susceptible isolates		One-factorial log. Reg.		Multi-factorial log. Reg.	
		n	%	n	%	OR	p	OR	p
Time period	2006–2011 (Ref.)	1345	74.64	457	25.36	1	–	1	–
	2012–2017	919	73.80	325	26.13	0.96	0.634	0.64	0.014
Age-group	Sow (Ref.)	24	63.16	14	36.84	1	–	1	–
	Piglet	102	72.86	38	27.14	1.57	0.246	2.46	0.173
	Nursery	578	79.5	149	20.5	2.26	0.019	3.71	0.045
	Fattening	33	66.0	17	34.0	1.13	0.782	1.76	0.414
Sampling site	GIT (Ref.)	1887	76.49	580	23.51	1	–	1	–
	GUT	188	56.29	146	43.71	0.40	< 0.0001	1.98	0.332
	others	189	77.14	56	22.86	1.04	0.818	1.19	0.527

In sows, 78.9%, and in all other age-groups, only 0.1–4.0% samples originated from the genito-urinary tract, so that age-dependent differences might also reflect sampling-site specific differences (Table 10). Indeed, in the data set containing 956 animals of known age-groups, similar findings were found for sows as for genito-urinary tract samples compared to the data pool of piglets, nursery and fattening pigs. In sows, the percentage of isolates resistant to ampicillin (63.2% vs. 77.8%, *p* = 0.05), colistin (3.3% vs. 17.9%, *p* = 0.5) and neomycin (0.0% vs. 14.1%, *p* = 0.01) were less and that of isolates resistant to enrofloxacin, (17.1% vs. 1.8%, *p* < 0.0001) was more frequent (data not shown).

### Temporal trends in AMR

**Figure 1:**
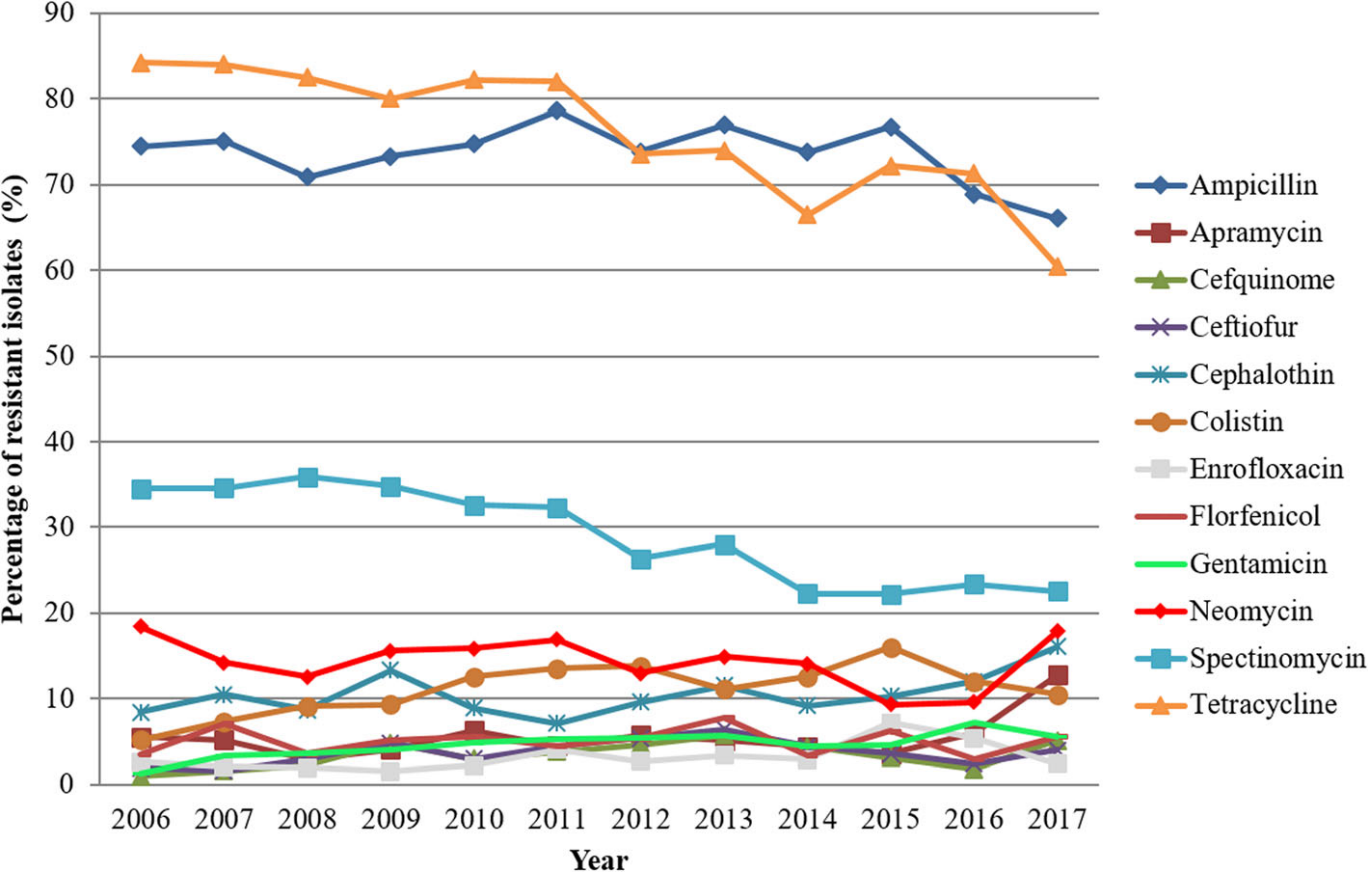
Time dependent development of resistant isolates in the years from 2006 to 2017

The relative frequency of resistant isolates in the different years from 2006 to 2017 is shown in Fig. 1 indicating several levels of AMR.

To address the hypothesis that reduction in antibiotic usage since 2011 had an influence on bacterial resistance development, the two six-year time periods from 2006 to 2011 and 2012–2017 were compared by means of the chi-square test as well as in one-factorial and multifactorial logistic regression models. A higher percentage of resistant isolates was found for all cephalosporins, colistin, enrofloxacin and gentamicin in the time period 2012–2017, while decreases were found for tetracycline, spectinomycin and neomycin. Regarding only isolates from the gastrointestinal tract, therefore excluding bias by different sampling sites resulted in similar trends for colistin, gentamicin, neomycin, spectinomycin and tetracycline (data not shown). Findings were specified by simple regression models comparing findings in single years to the allocation base 2011 (data not shown).

A multifactorial logistic regression model was performed for comparing both time periods, simultaneously taking factors age-group and sampling site into account. While differences in frequencies of resistant isolates with respect to cefquinome, ceftiofur and colistin were due to age-group (Additional files 1b-c, e), differences with regard to neomycin, spectinomycin and tetracycline were related to the time periods (Tables 2, 3 and 4).

Differences with respect to ampicillin were significantly due to age-group and time (Table 5). The sampling site had no significant influence in this model.

The percentage of neomycin- (18.4 to 17.9%), spectinomycin- (34.5 to 22.6%), tetracycline- (84.2 to 60.5%) and ampicillin-resistant (74.5 to 66.1%) isolates decreased from 2006 to 2017 (Tables 6, 7, 8 and 9). The development of frequencies of resistant isolates to other antimicrobials throughout the years are shown in Additional file 2a-h. With respect to epidemiological cut-offs, similar trends were observed. While time-dependent decreases were found for frequencies of resistant isolates with respect to neomycin, spectinomycin and tetracycline, additionally, a significant age-group related increase in enrofloxacin-resistant isolates (15.2 to 16.1%) from 2006 to 2017 was observed (*p* = 0.002) (Data not shown).

**Table 6:** Temporal development in AMR for neomycin

	Number of isolates with MIC values (mg/ml) of…							S		I		R	
	4	16	32	64	n	MIC _50_	MIC _90_	n	%	n	%	n	%
Year													
2006	242	11	25	32	310	4.00	64.00	242	78.06	11	3.55	57	18.39
2007	319	8	24	30	381	4.00	32.00	319	83.73	8	2.10	54	14.17
2008	266	4	15	24	309	4.00	32.00	266	86.08	4	1.29	39	12.62
2009	227	1	9	33	270	4.00	64.00	227	84.07	1	0.37	42	15.56
2010	224	3	15	28	270	4.00	64.00	224	82.96	3	1.11	43	15.93
2011	218	3	20	25	266	4.00	32.00	218	81.95	3	1.13	45	16.92
2012	227	.	2	32	261	4.00	64.00	227	86.97	.	.	34	13.03
2013	251	1	13	31	296	4.00	64.00	251	84.80	1	0.34	44	14.86
2014	175	2	9	20	206	4.00	32.00	175	84.95	2	0.97	29	14.08
2015	175	1	3	15	194	4.00	4.00	175	90.21	1	0.52	18	9.28
2016	145	6	3	13	167	4.00	16.00	145	86.83	6	3.59	16	9.58
2017	31	1	3	4	39	4.00	64.00	31	79.49	1	2.56	7	17.95
Total	2500	41	141	287	2969	4.00	32.00	2500	84.20	41	1.38	428	14.42

**Table 7:** Temporal development in AMR for spectinomycin

	Number of isolates with MIC values (mg/ml) of…									S		I		R	
	4	16	32	64	128	256	n	MIC_50_	MIC_90_	n	%	n	%	n	%
Year															
2006	17	94	52	40	32	75	310	32.00	256.00	163	52.58	40	12.90	107	34.52
2007	2	106	87	54	41	91	381	32.00	256.00	195	51.18	54	14.17	132	34.65
2008	3	105	51	39	32	79	309	32.00	256.00	159	51.46	39	12.62	111	35.92
2009	1	81	65	29	25	69	270	32.00	256.00	147	54.44	29	10.74	94	34.81
2010	.	79	66	37	20	68	270	32.00	256.00	145	53.70	37	13.70	88	32.59
2011	4	97	53	26	41	45	266	32.00	256.00	154	57.89	26	9.77	86	32.33
2012	3	80	66	43	26	43	261	32.00	256.00	149	57.09	43	16.48	69	26.44
2013	3	120	57	33	32	51	296	32.00	256.00	180	60.81	33	11.15	83	28.04
2014	3	93	47	17	18	28	206	32.00	256.00	143	69.42	17	8.25	46	22.33
2015	12	71	47	21	10	33	194	32.00	256.00	130	67.01	21	10.82	43	22.16
2016	5	72	35	16	15	24	167	32.00	256.00	112	67.07	16	9.58	39	23.35
2017	.	52	31	13	24	4	124	32.00	128.00	83	66.94	13	10.48	28	22.58
Total	53	1050	657	368	316	610	3054	32.00	256.00	1760	57.63	368	12.05	926	30.32

**Table 8:** Temporal development in AMR for tetracycline

	Number of isolates with MIC values (mg/ml) of…											S		I		R	
	0.25	0.5	1	2	4	8	16	32	n	MIC_50_	MIC_90_	n	%	n	%	n	%
Year																	
2006	.	26	.	20	2	1	1	260	310	32.00	32.00	48	15.48	1	0.32	261	84.19
2007	.	20	.	38	2	1	.	320	381	32.00	32.00	60	15.75	1	0.26	320	83.99
2008	.	8	.	37	8	1	3	252	309	32.00	32.00	53	17.15	1	0.32	255	82.52
2009	.	3	.	43	6	2	2	214	270	32.00	32.00	52	19.26	2	0.74	216	80.00
2010	.	5	.	35	7	1	2	220	270	32.00	32.00	47	17.41	1	0.37	222	82.22
2011	.	21	.	21	4	2	1	217	266	32.00	32.00	46	17.29	2	0.75	218	81.95
2012	.	9	.	52	8	.	.	192	261	32.00	32.00	69	26.44	.	.	192	73.56
2013	.	18	.	53	4	2	2	217	296	32.00	32.00	75	25.34	2	0.68	219	73.99
2014	1	5	17	41	4	1	.	137	206	32.00	32.00	68	33.01	1	0.49	137	66.50
2015	1	.	5	46	1	1	.	140	194	32.00	32.00	53	27.32	1	0.52	140	72.16
2016	.	.	7	39	2	.	1	118	167	32.00	32.00	48	28.74	.	.	119	71.26
2017	.	.	13	36	.	.	51	24	124	16.00	32.00	49	39.52	.	.	75	60.48
Total	2	115	42	461	48	12	63	2311	3054	32.00	32.00	668	21.87	12	0.39	2374	77.73

**Table 9:** Temporal development in AMR for ampicillin

	Number of isolates with MIC values (mg/ml) of…												S		I		R	
	0.25	0.5	1	2	4	8	16	32	64	n	MIC _50_	MIC _90_	n	%	n	%	n	%
Year																		
2006	.	2	15	46	13	2	1	1	230	310	64	64	78	25.16	1	0.32	231	74.52
2007	.	.	7	55	29	4	.	4	282	381	64	64	95	24.93	.	.	286	75.07
2008	.	.	2	50	30	7	1	.	219	309	64	64	89	28.80	1	0.32	219	70.87
2009	.	.	4	28	33	6	1	1	197	270	64	64	71	26.30	1	0.37	198	73.33
2010	.	.	5	32	30	1	.	1	201	270	64	64	68	25.19	.	.	202	74.81
2011	.	.	10	33	13	.	1	.	209	266	64	64	56	21.05	1	0.38	209	78.57
2012	.	.	1	35	27	4	1	2	191	261	64	64	67	25.67	1	0.38	193	73.95
2013	.	.	2	19	42	5	.	1	227	296	64	64	68	22.97	.	.	228	77.03
2014	1	.	4	23	22	3	1	3	149	206	64	64	53	25.73	1	0.49	152	73.79
2015	.	.	1	17	24	2	1	.	149	194	64	64	44	22.68	1	0.52	149	76.80
2016	.	.	2	27	19	3	1	1	114	167	64	64	51	30.54	1	0.60	115	68.86
2017	.	.	.	25	16	1	.	1	81	124	64	64	42	33.87	.	.	82	66.13
Total	1	2	53	390	298	38	8	15	2249	3054	64	64	782	25.61	8	0.26	2264	74.13

Tables 2, 3, 4 and 5 Multifactorial logistic regression analysis with fixed effect time period and factors “age-group” and “sampling site” with respect to neomycin (2), spectinomycin (3), tetracycline (4) and ampicillin resistance (5). Reference categories for the logistic regression method (time period 2006–2011, sow, gastrointestinal tract) are highlighted in bold and indicated by “Ref.”. One-factorial log. Reg.: One-factorial logistic regression model, Multi-factorial log.reg.: Multi-factorial logistic regression model, OR: Point estimate /Odds ratio, p: *p*-value of the Wald test, n: absolute number of isolates, %: percentage of isolates.

## Discussion

In this study, based on routine diagnostic data from swine farms in a swine-dense region in North Western Germany, antimicrobial resistances were analysed for *E. coli* isolated in a diagnostic institute between 2006 and 2017. All isolates originated from diseased animals and the data set was restricted to one isolate from one farm per year.

In general, studies dealing with bacterial resistance focus on either i) analysis of routine data as was the case in this study, ii) standardised experiments and epidemiological studies or iii) resistance monitoring following a statistically valid sampling programme [29, 30]. Secondary data from daily routine as used in the present analyses are prone to different types of bias. This is mainly due to the non-standardised and non-representative sampling of diseased animals and the lack of metadata, which leads to a confounding bias in the results.

Samples originated from the North Western part of Germany might therefore not be representative of the whole of Germany and therefore might be prone to a selection bias. Nevertheless, more than half of the German swine population is located in this region, so that findings are of importance for the whole country. Moreover, regional differences in antimicrobial use were not recorded for Germany [20, 21].

Due to high standardisation of the routine susceptibility testing of isolates throughout the years, an information bias can be ignored in this study. Although *E. coli* strains analysed in this study were considered to be responsible for the disease picture, a clear microbiological differentiation between commensal and pathogenic *E. coli* strains is not possible [31, 32]. In this study, assessing analysed *E. coli* strains as pathogenic was based on clinical and pathological observations as well as quantification of growth and phenotype of cultured *E. coli* strains. The *E. coli* selection and testing procedure was carried out in one accredited laboratory and by only two experienced scientists during the entire study period. This is in contrast to other national and international resistance monitoring programmes collecting preselected isolates from various laboratoriess.

The most important drawback of this study was the lack of meta-data, which mask potential confounding effects. It is known that AMR is influenced by a variety of factors present on farms including endemic infections, cleaning and disinfection protocols, safety requirements for farmers and others [11]. These data cannot be allocated to the respective samples. In this study, comparison of one- and multifactorial regression analysis results revealed these confounding effects by only taking the factors time period, age groups and sampling site into account (Tables 2, 3, 4 and 5). As an example, confounding becomes obvious regarding ampicillin: a simple comparison of the sampling site revealed significant differences in susceptibility of isolates originating from the genito-urinary and the gastrointestinal tract. This effect is diminished when also taking the time period into account in a multifactorial logistic regression model (Table 5). Overall, this problem of residual confounding cannot be eliminated completely in the context of routine data evaluation. In field studies, only a low number of pigs are sampled on one farm and only a small number of bacterial colonies are tested, which might not been representative of the species population on the respective farm [33].

Nevertheless, in spite of the aforementioned drawbacks of this field study, finding significant trends in resistance development from a routine data pool even under these conditions, hints at relevant phenomena of practical impact.

As all MICs have already been determined in routine diagnostics and form the basis for the decision regarding respective antimicrobial substances for animal treatment, our data evaluation was based on the clinical cut-off definition. These clinical break-points are determined by achievable tissue concentrations after treatment with the respective substance [34, 35].

Assessing susceptibility of isolates in accordance with epidemiological cut-offs (www.eucast.org, access 8/8/2019) resulted in slightly different frequencies of resistant isolates, which were in all cases higher or equal to those assessed by clinical cut-offs (Table 1). This indicates that isolates originate from an artificial environment in domestic pigs exposed to other factors other than wild-type isolates. It must be assumed that they had been targeted by antibiotic treatment. This means that the bacterial population had already undergone a selective pressure.

It was hypothesised that a reduction in antibiotic usage in swine populations in the North Western part of Germany would be reflected by a reduction in frequencies of resistant isolates over time. Multifactorial logistic regression models and analysis of a data subset containing information about organ sampling site and age-group revealed that only some significant changes in frequencies of resistant isolates can be assigned to the time period. Some findings might reflect the usage of different antimicrobial substances in different age-groups due to the respective dominant disease pictures. This was most obvious when comparing samples from the gastrointestinal and the genitourinary tract. For the substances which are widely used for oral treatment of gastrointestinal tract disorders and have a low tissue penetration, frequencies of resistant isolates were lower in the genito-urinary tract. Gastrointestinal disorders in sows are rare and therefore not often targeted by antimicrobial treatment. For enrofloxacin and florfenicol with a high tissue penetration, which are often used in sows for treating bacterial infections of the genito-urinary and respiratory tract, resistant isolates were more frequent [36].

In the resistance monitoring programme GERMAP [24] in pigs with gastrointestinal disorders, frequencies of resistant *E. coli* isolates were comparable to those reported in our study: tetracyclines (69–73%), ampicillin (67–74%) and gentamicin (5–12%). MIC_90_ of both German sampling pools slightly differed with higher values for apramycin and lower values for enrofloxacin in 2012 in our study compared to the nursery pig evaluation in GERMAP 2015 [24]. In GERMAP, isolates originate from different geographical regions, but are preselected by different laboratories. Meta-data are missing as in most existing national and international monitoring programmes. In spite of these restrictions, it has to be taken into account that differences in frequencies of resistant isolates between different studies are partly due to these methodological differences and therefore have to be interpreted with caution [37].

According to literature studies, our data differ to other regions of the world. In a study in 2018 on commensal *E. coli* isolates from healthy slaughter pigs in Australia, frequencies of resistant isolates (ampicillin ~ 60.2%, tetracycline 68.2%, ciprofloxacin 1%, gentamicin and ceftiofur 0%) were lower than in our German sampling [38], which is mainly due to the fact that only healthy slaughter pigs were sampled in this previous study. A recent study in Estonia revealed lower percentages of tetracycline-resistant *E. coli* isolates in healthy swine (32.5%) compared to diseased swine (60.2%) as well [39]. The comparison of haemolytic *E. coli* pathotypes isolated from diarrhoeic piglets in the USA revealed marked differences in susceptibility between ETEC and non-ETEC with respect to enrofloxacin (58.2% vs. 5.0%) and gentamicin (32.7% vs. 7.5%) [40]. Overall, strains were highly resistant to oxytetracycline (91.6%) and ampicillin (75.8%) in this study.

Although in our study no simultaneous data on respective farms for AMU and AMR were evaluated, diagnostic findings in most swine dense regions in Germany might reflect the effects of efforts to reduce antibacterial resistances. In general, low frequencies with less than 15% of resistant isolates were found for colistin, neomycin, cefquinome, ceftiofur, cephalothin, gentamicin, enrofloxacin, florfenicol and apramycin, while higher frequencies were found for ampicillin, tetracycline and spectinomycin. The time-dependent analysis of antimicrobial resistances in *E. coli* reported in this study resulted in a significant decrease in the frequency of isolates resistant to tetracycline to the aminoglycosid antibiotics neomycin and spectinomycin in the multivariate logistic regression model. These decreases are paralleled by a decrease in sales data for these substances in Germany since 2012 [41] as well as by a reduction in antibiotic usage in livestock since 2011, which was paralleled by monitoring projects and the implementation of the Second Version of Guidelines on the Prudent Use of Veterinary Antimicrobial Drugs presented by the Federal Veterinary Surgeons’ Association [21, 22, 42]. This corresponded to the reduction in the number of administered daily doses in the same observation period. Significant changes in treatment frequencies were already recorded as results within the German VetCab project from the first half of 2011 to the second half of 2014 in piglets (decrease from 3.8 to 1.7) and fattening pigs (5.1–0.7), while treatment frequency in weaners was highly variable and in sows remained constantly low [21]. Since the start of antibiotic usage records which have been required 2014, the median treatment frequency for weaners has been reduced from 4.79 to 3.02 and for fatteners from 1.19 to 0.3 [23, 43, 44]. Efforts to reduce antibiotic usage in livestock at working levels were accompanied by a radical strengthening of the veterinary control stipulated in the German Pharmaceutical Act in 2014 [25]. Treatment frequencies with respect to particular antibiotic substances reported in VetCab [22], but also continuously in the German Quality assurance database (www. q-s.de/softwareplattform/en/, access 10/11/2019) show similar trends as the sales data for antibiotics drugs. The highest reduction rate in sales data from 2011 to 2017 was achieved for tetracyclines by more than 66%. Aminoglycosid antibiotics were reduced by more than 37% in the same time-period [16]. In contrast to that, sales data of fluorchinolones belonging to the Highest Priority Critically Important Antimicrobials varied between the years, so that in fact. no reduction was achieved. This might be reflected by an age-group related increase in the percentage of enrofloxacin-resistant isolates in sows. Nevertheless, the fact that samples from the genito-urinary tract from sows nearly halved in the time-period 2012–2017 might be due to a better health status of sows by improved vaccination and gilt acclimatisation protocols. In addition, higher numbers of piglets born alive due to advanced breeding resulting in a higher reproductive performance of sows, has demanded excellent management in the recent years. Increased standards in pig production require more specialised farmers who are able to perpetuate a high health status in their herd. The generally higher health status in sows might be reflected by less sampling and an age- and time-dependent reduction in ampicillin-resistant isolates. This development is in parallel to a reduction in sales data for penicillins by 49% in the same time period [16]. Alternatively individual parenteral treatment of sows with fluorchinolones might be chosen as a treatment strategy by farmers.

In general, the relationship between antibiotic treatment and occurrence of resistant isolates has been shown in livestock animals [45]. Recently, a positive relationship between oral administration of chlortetracycline in nursery pigs and the probability of occurrence of *E. coli* isolates resistant to chlortetracycline and ceftiofur has been described [46]. In Australia, the very low level of non-susceptibility to critically important antimicrobials such as third-generation cephalosporins and fluoroquinolones in healthy slaughter pigs might be attributed to the fact that fluoroquinolones are not legally available for food-producing animals in this continent [38]. In 2006/2007, a limited number of 43 *E. coli* isolates also from healthy swine in Argentina were examined for minimal inhibitory concentration distribution [47]. The authors attributed the levels of non-susceptible isolates to those drugs used most commonly in pig production (ampicillin ~ 77%, tetracycline 88%, florfenicol 98%, gentamicin 5%, enrofloxacin 2%) [47].

A literature systematic review based on thirteen relevant articles revealed a relationship between AMR pattern in herds and dosage, route of administration and frequency of AMU. Several additional factors had a statistically significant influence on AMR on herd level, as space allowance, cleanliness, time span between sampling and treatment and distance to another farm [48]. Higher odds ratios were found for resistance of *E. coli* to quinolones, aminoglycosides and tetracycline in herds with oral administration of antimicrobials [7]. All published studies linking AMU to AMR revealed that research must focus on the effects of specific management practices more than only on AMU. Understanding resistance mechanisms, their distribution among bacterial populations and environmental factors triggering their occurrence are indispensable. Not only antimicrobial substances produce a selective pressure towards a more resistant bacterial community, but also several other substances which are present in a farm environment. Naturally occurring antimicrobial substances can be produced by bacterial species, especially in a tight bacterial community as in biofilms, e.g. in slurry, water or feeding pipes. At these localisations, a high density of bacterial cells enables horizontal gene transfer as an additional factor for the spread of resistant bacteria in the environment [49].

A general bacterial stress response or expression of multi-drug resistance pumps following exposure to disinfectants, can also trigger development of resistance to antimicrobials [50]. This effect was also found after bacterial exposure to specific nutritional elements in feed, e.g. zinc and copper [51]. Zinc is used in high concentrations (1000–3000 ppm) for treating diarrohea or enterotoxaemia caused by pathogenic *E. coli* in nursery pigs [52].

In general, to link antibiotic usage to resistance development at farm level, fundamental data are necessary, which are lacking in this study. Antibiotic consumption data have to be recorded at farm level, including name and galenic product, number of treated animals, indication and dosage [24]. Sample size and sampling sites for susceptibility testing in an adequate number of isolates should be determined following statistical recommendations. For future analyses, if efforts to reduce antibiotic usage are effective to reduce resistant bacterial isolates, the evaluation of more epidemiological data as well as case-control studies are necessary. The present study can be considered as a first step in analysing trends in resistance development in the region with highest pig density and highest consumption of antimicrobial substances in livestock animals in Germany.

## Conclusion

*E.coli* is a well accepted sentinel for antimicrobial resistance monitoring in livestock. In a North Western part of Germany, the effects of age-group, organ sampling sites and the time-period on the frequency of antimicrobial resistances were found within the years 2011 to 2017. The percentage of tetracycline-, spectinomycin- and neomycin-resistant isolates decreased significantly in a time-dependent manner. This observation is in parallel with a reduction in usage of these antibiotic substances in Germany. In summary, the efficiency of antibiotic drugs for the future can be preserved if prescription and usage of antimicrobial substances are further controlled and monitored in human and veterinary medicine and are in part replaced by alternative treatment approaches. Simultaneously, monitoring of bacterial resistances in combination with recording of data on exposure of the respective strains to factors triggering development of antimicrobial resistance will pave the way for new strategies to decrease the development of antibacterial resistance.

## Methods

### Sample collection, isolation of bacterial strains and susceptibility testing

From 1 January 2006 to 31 December 2017, MIC values from 3054 *E. coli* isolates from diseased pigs sent for routine diagnostics or sampled on-farm were evaluated. Within 1 year, only the first *E. coli* isolate from a respective farm was included in the data set. The majority of isolates originated from different organs of diseased swine, which had been necropsied for diagnostic reasons. All other isolates originated from samples which had been taken by veterinarians from diseased animals on swine farms and sent for diagnostics. In total, isolates originated from 2161 farms in a swine dense region in North Western Germany. The majority, that of 95.8% of the farms, were located in three neighbouring zones. Identification numbers were allocated to individual samples and the information related to the sample, as origin, sampling site, date of sampling, the bacteria isolated and their MIC as well as qualitative assessment of resistance. In a subset of data, also information about the age group was available (*n* = 956). Distribution of sampling sites among the age groups is shown in Table 10.

**Table 10:** Distribution of *E. coli* isolates originating from different sampling sites and age groups

Sampling site	Suckling piglets		Nursery pigs		Fatteners		Sows		All samples^a^	
	n	%	n	%	n	%	n	%	n	%
Jejunum	64	45.7	511	70.2	30	60.0	3	7.9	1192	39.0
Feces	1	0.7	0	0	1	2.0	0	0	998	32.7
Mesenteric lymph nodes	20	14.3	184	25.3	11	22.0	0	0	283	9.3
Genito-urinary tract	1	0.7	1	0.1	2	4.0	30	78.9	336	11.0
Other organs	54	38.6	32	4.4	6	12.0	5	13.2	245	8.0
Total	140	100	728	100	50	100	38	100	3054	100

^a^including samples from pigs without information about age-group

*E. coli* was identified by standard bacteriological cultivation and biochemical tests. In total, 39% (1192) of the *E. coli* strains were isolated from the jejunum, 32.7% (998) from faeces and 9.3% (283) from the mesenteric lymph nodes. Eleven % (336) of isolates originated from the genito-urinary tract and 8% (245) from other organs.

Isolates from sows (4%) originated mainly from the genito-urinary tract (79%). The highest percentage of isolates was collected from nursery pigs (76.1%), followed by suckling piglets (14.6%) and fatteners (5.2%). In these age-groups, most isolates were from the gastro-intestinal tract (suckling piglets: 61%, nursery piglets: 96%, fatteners 84%). No commensal *E. coli* were included in the evaluation. Pathogenic potential of isolates was assessed by clinical and pathomorphological findings, the quantity of growth in primary culture and the phenotype of the colonies.

### Antimicrobial susceptibility testing

Seventeen antimicrobial agents of different concentrations were included in antimicrobial susceptibility testing of bacterial strains following the recent CLSI manuals [53, 54] in a commercial microtiter plate assay (Sensititre® NLV 39, TREK Diagnostic Systems Ltd., Cleveland, USA) with a standardised layout for livestock animals [55]: ampicillin (0.12–32 μg/mL), apramycin (8–32 μg/mL), ceftiofur (0.12–8 μg/mL), cefquinome (1–8 μg/mL), cephalothin (1–16 μg/mL), clindamycin (0.25–4 μg/mL), colistin (0.5–4 μg/mL), erythromycin (0.12–4 μg/mL), enrofloxacin (0.03–2 μg/mL), florfenicol (1–8 μg/mL), gentamicin (0.25–8 μg/mL), neomycin (8-32 μg/mL), penicillin G (0.06–16 μg/mL), spectinomycin (4–64 μg/mL), tetracycline (0.12–8 μg/mL), tiamulin (8–32 μg/mL), tilmicosin (1–32 μg/mL). Data for penicillin G, erythromycin, clindamycin, tiamulin and tilmicosin were not included in the evaluation for its natural resistance to these substances.

The *E. coli* reference strain ATCC 25922 was used as control strain. Colony material of the respective isolate was suspended in 5 mL NaCl and optical density was adjusted to 0.5 in Mc Farland broth (Becton, Dickinson and Company, Maryland, USA) measured in a densitometer (bioMérieux Marcy l’Etoile, Marcy l’Etoile, France). Optical density of 0.5 corresponds to 10^6^–10^8^ CFU/mL. Ten microliter of the suspension was mixed with 10 mL of sterile Müller-Hinton-Bouillon and 50 μL of the inoculum were transferred to each well of a commercially available microtiter plate (Sensititre® NLV 39, TREK Diagnostic Systems Ltd., Cleveland, USA). Different wells were coated with different concentrations of antimicrobial substances in two-fold dilutions in a range overlapping MICs reflecting resistance and intermediate susceptibility in accordance with the cut-off of the test organism as well as the Clinical and Laboratory Standards Institute (CLSI) cut-offs. The microtiter plate was incubated at 36 °C ± 1 °C for 18–24 h. Results were electronically recorded and printed out with the Sensitouch device (Sensititre, Cleveland, USA). Susceptibility and resistance of bacterial isolates were assessed with respect to clinical breakpoints of the CLSI guidelines [53, 56]. Bacterial isolates were categorised as “susceptible” (s), “intermediate” (i) or “resistant” (r). During the evaluation period 2006–2017, all testings were performed by the same two experienced persons in the same accredited laboratory following the respective Standard Operating Procedures. For the years evaluated, always the same cut-offs were used for assessing the resistance patterns. If no clinical cut-offs were available, other published cut-offs were used. For ampicillin, cephalothin, gentamicin and tetracycline, neither animal-specific nor pathogen-specific cut-offs were available. Therefore, the cut-offs for *E. coli* isolates from human were used. Cut-offs were as follows (susceptible/ intermediate/ resistant, concentrations in μg/mL): ampicillin: ≤8 / 16 / ≥32 [57]; cephalothin: ≤8 / 16 / ≥32 [56]; gentamicin: ≤4 / 8 / ≥16 [57] and tetracycline: ≤4 / 8 / ≥16 [57]. The MICs for ceftiofur (≤2 / 4 / ≥8) [57], enrofloxacin (≤0.25 / 0.5–1 / ≥2) [57], florfenicol (≤2 / 4 / ≥8) [53] and spectinomycin (≤32 / 64 / ≥128) [53] were validated for animals. The MICs for apramycin (≤16 / - / ≥32), cefquinome (≤2 / 4 / ≥8), colistin (≤0.5 / 1–2 / ≥4) and neomycin (≤8 / 16 / ≥32) were based on DIN 58940 for humans [58]. For assessing an epidemiological impact of strains originating from diseased animals, an additional evaluation was performed based on available current epidemiological cut-offs for *E. coli* [59]. The cut-offs are shown in Table 11.

**Table 11:** Clinical and epidemiological cut-offs

	Clinical cut-off (μg/mL) ≤	Reference for clinical cut-off	Epidemiological cut-off (μg/mL) ≤
Ampicillin	8	Human [57]	8
Apramycin	16	Human [58]	
Ceftiofur	2	Animal [57]	1
Cefquinome	2	Human [58]	
Cephalothin	8	Human [56]	32
Colistin	0.5	Human [58]	2
Enrofloxacin	0.25	Animal [57]	0.125
Florfenicol	2	Animal [53]	16
Gentamicin	4	Human [57]	2
Neomycin	8	Human [58]	8
Spectinomycin	32	Animal [53]	64
Tetracycline	4	Human [57]	8

Clinical and epidemiological cut-offs used in this study for assessing MIC in *E. coli* isolates from diseased pigs. Epidemiological cut-off (μg/mL): European Committee on Antimicrobial Susceptibility Testing. Data from the EUCAST MIC distribution website, last accessed 05.08.2019, http://www.eucast.org).

### Data management

All data were registered by means of the laboratory information system LabControl, Version 2002, Ticono-Software, Hannover and exported to Excel, Version 2010 (Microsoft Corporation, Albuquerque, USA). Statistical analyses were performed via SAS, version 9.4 (SAS Institute, NC, USA).

Different sampling site descriptions existing in the database were summarised to three localisations as the gastrointestinal tract containing jejunum, faeces and mesenteric lymph nodes, genito-urinary tract and other organs (Table 10).

MIC distributions of each antimicrobial agent were visualised using bar graphs showing the relative frequencies of susceptible, intermediate and resistant isolates. MIC_50_ and MIC_90_-values were calculated, corresponding to the median and the 90%-quantile of the sampling pool, respectively [60]. Isolates were categorised as “susceptible” and “resistant”. To address the hypothesis of an impact of reduction in AMU and stricter legislation since 2011 with respect to prescription of antimicrobials on frequencies of resistant isolates, the final evaluation of data was performed by a comparison of the two time periods 2006–2011 and 2012–2017. Frequencies were compared by means of the chi-square test and logistic regression models.

To visualise the temporal AMR development per antimicrobial agent, graphs were generated showing the relative percentage of resistant isolates over time (Fig. 1).

The chi-square test was used for the comparing AMR frequencies from different sampling sites, age-groups and time-periods. Subsequently, simple logistic regression models were calculated to further identify major differences between groups. Finally, a multifactorial logistic regression model was performed to analyse the impact of age-group and sampling site on temporal changes in frequencies of resistant isolates. The level of significance for all statistical models was set at 0.05 without any adjustment for multiple testing.

## Supplementary information

**Additional file 1.** Multifactorial logistic regression analysis with fixed effect time period and factors “age-group” and “sampling site” with respect to apramycin resistance (1a), cefquinome resistance (1b), ceftiofur resistance (1c), cephalothin resistance (1d), colistin resistance (1e), enrofloxacin resistance (1f), florfenicol resistance (1 g) and gentamicin resistance (1 h).

**Additional file 2.** Temporal development in AMR for apramycin (1a), cefquinome (1b), ceftiofur (1c), cephalothin (1d), colistin (1e), enrofloxacin (1f), florfenicol (1 g) and gentamicin (1 h).

## Data Availability

The datasets used and/or analysed during the current study are available from the corresponding author on reasonable request.

## Abbreviations

AMR: Antimicrobial resistance
AMU: Antimicrobial use
ATCC: American Type Culture Collection
CFU/mL: Colony-forming unit per millilitre
CLSI: Clinical and Laboratory Standards Institute
DIMDI-Ordinance: Ordinance on the Database-supported Information System on Medical Devices of the German Institute for Medical Documentation and Information
DNA: Deoxyribonucleic acid
*E. coli*: *Escherichia coli*
e.g.: exempli gratia
EARSS: European Antimicrobial Resistance Surveillance System
ETEC: Enterotoxic *Escherichia coli*
GERMAP: German Programme for surveillance of antimicrobial consumption and resistance in bacteria
GIT: Gastrointestinal tract
GLASS: Global Antimicrobial Resistance Surveillance System
GUT: Genito-urinary tract
i: intermediate
mg/L: milligramme per litre
mg/mL: milligramme per millilitre
mg/PCU: milligramme per Population Correction Units
MIC: Minimal inhibitory concentration
mL: millilitre
n: number
NaCl: Sodium chloride
OIE: Office International des Epizooties
ppm: parts per million
R [%]: Relative frequency
r: resistant
s: susceptible
WHO: World Health Organization
μg/kg: Microgramme per kilogramme
μg/mL: Microgramme per millilitre
μL: Microlitre
