# Supplementary material for: Phenotypic antimicrobial resistance in Escherichia coli strains isolated from swine husbandries in North Western Germany – temporal patterns in samples from laboratory practice from 2006 to 2017
Source: BMC Vet Res. 2020 Feb 3;16:37. doi: 10.1186/s12917-020-2268-z (PMC6998819; doi:10.1186/s12917-020-2268-z)
Supplement: Supplementary file 1 — Additional file 1. Multifactorial logistic regression analysis with fixed effect time period and factors “age-group” and “sampling site” with respect to apramycin resistance (1a), cefquinome resistance (1b), ceftiofur resistance (1c), cephalothin resistance (1d), colistin resistance (1e), enrofloxacin resistance (1f), florfenicol resistance (1 g) and gentamicin resistance (1 h). [file 12917_2020_2268_MOESM1_ESM.docx]

**Additional file 1a**. Apramycin resistance

|  |  | Resistant isolates | | Susceptible isolates | | One-factorial log. reg. | | Multi-factorial log. reg. | |
| --- | --- | --- | --- | --- | --- | --- | --- | --- | --- |
|  |  | n | % | n | % | OR | p | OR | p |
| **Time period** | **2006-2011** (Ref.) | 86 | 4.76 | 1720 | 95.24 | 1 | - | 1 | - |
|  | 2012-2017 | 61 | 5.25 | 1102 | 94.75 | 1.10 | 0.554 | 1.52 | 0.265 |
|  |  |  |  |  |  |  |  |  |  |
| **Age-group** | **Sow** (Ref.) | 0 | 0 | 35 | 100 | 1 | - | 1 | - |
|  | Piglet | 4 | 3.03 | 128 | 96.97 | - | 0.957 | - | 0.960 |
|  | Nursery | 35 | 5.09 | 653 | 94.91 | - | 0.955 | - | 0.9959 |
|  | Fattening | 2 | 4.35 | 44 | 95.65 | - | 0.55 | - | 0.959 |
|  |  |  |  |  |  |  |  |  |  |
| **Sampling site** | **GIT** (Ref.) | 129 | 5.38 | 2266 | 94.62 | 1 | - | 1 | - |
|  | GUT | 9 | 2.73 | 321 | 97.27 | 0.49 | 0.043 | <0.001 | 0.962 |
|  | others | 9 | 3.72 | 233 | 96.28 | 0.68 | 0.271 | 0.21 | 0.138 |

A**dditional file 1b.** Cefquinome resistance

|  |  | Resistant isolates | | Susceptible isolates | | One-factorial log. reg. | | Multi-factorial log. reg. | |
| --- | --- | --- | --- | --- | --- | --- | --- | --- | --- |
|  |  | n | % | n | % | OR | p | OR | p |
| **Time period** | **2006-2011** (Ref.) | 47 | 2.61 | 1751 | 97.39 | 1 | - | 1 | - |
|  | 2012-2017 | 49 | 4.23 | 1110 | 95.77 | 1.65 | 0.017 | 0.99 | 0.988 |
|  |  |  |  |  |  |  |  |  |  |
| **Age-group** | **Sow** (Ref.) | 1 | 2.86 | 34 | 97.14 | 1 | - | 1 | - |
|  | Piglet | 11 | 8.33 | 121 | 91.67 | 3.09 | 0.288 | 3.53 | 0.471 |
|  | Nursery | 12 | 1.75 | 674 | 98.25 | 0.61 | 0.634 | 0.66 | 0.813 |
|  | Fattening | 3 | 6.67 | 42 | 93.33 | 2.43 | 0.451 | 2.66 | 0.589 |
|  |  |  |  |  |  |  |  |  |  |
| **Sampling site** | **GIT** (Ref.) | 70 | 2.93 | 2318 | 97.07 | 1 | - | 1 | - |
|  | GUT | 5 | 1.52 | 324 | 98.48 | 0.51 | 0.150 | 1.13 | 0.945 |
|  | others | 21 | 8.75 | 129 | 91.25 | 3.18 | <0.0001 | 0.86 | 0.788 |

**Additional file 1c**. Ceftiofur resistance

|  |  | Resistant isolates | | Susceptible isolates | | One-factorial log. reg. | | Multi-factorial log. reg. | |
| --- | --- | --- | --- | --- | --- | --- | --- | --- | --- |
|  |  | n | % | n | % | OR | p | OR | p |
| **Time period** | **2006-2011** (Ref.) | 54 | 3 | 1745 | 97 | 1 | - | 1 | - |
|  | 2012-2017 | 58 | 4.65 | 1189 | 95.35 | 1.58 | 0.018 | 0.83 | 0.639 |
|  |  |  |  |  |  |  |  |  |  |
| **Age-group** | **Sow** (Ref.) | 1 | 2.63 | 37 | 97.37 | 1 | - | 1 | - |
|  | Piglet | 12 | 8.57 | 128 | 91.43 | 3.47 | 0.240 | 3.29 | 0.469 |
|  | Nursery | 16 | 2.2 | 710 | 97.8 | 0.83 | 0.862 | 0.80 | 0.892 |
|  | Fattening | 3 | 6.12 | 46 | 93.88 | 2.41 | 0.454 | 2.30 | 0.623 |
|  |  |  |  |  |  |  |  |  |  |
| **Sampling site** | **GIT** (Ref.) | 79 | 32 | 2388 | 96.8 | 1 | - | 1 | - |
|  | GUT | 6 | 1.79 | 329 | 98.21 | 0.55 | 0.164 | 0.97 | 0.986 |
|  | others | 27 | 11.07 | 217 | 88.93 | 3.76 | <0.0001 | 1.06 | 0.912 |

**Additional file 1d**. Cephalothin resistance

|  |  | Resistant isolates | | Susceptible isolates | | One-factorial log. reg. | | Multi-factorial log. reg. | |
| --- | --- | --- | --- | --- | --- | --- | --- | --- | --- |
|  |  | n | % | n | % | OR | p | OR | p |
| **Time period** | **2006-2011** (Ref.) | 172 | 13.77 | 1077 | 86.23 | 1 | - | 1 | - |
|  | 2012-2017 | 138 | 17.27 | 661 | 82.73 | 1.31 | 0.031 | 1.34 | 0.287 |
|  |  |  |  |  |  |  |  |  |  |
| **Age-group** | **Sow** (Ref.) | 6 | 28.57 | 15 | 71.43 | 1 | - | 1 | - |
|  | Piglet | 17 | 22.88 | 60 | 77.92 | 0.71 | 0.535 | 1.90 | 0.653 |
|  | Nursery | 55 | 11.65 | 417 | 88.35 | 0.33 | 0.028 | 0.93 | 0.956 |
|  | Fattening | 6 | 14.63 | 35 | 85.37 | 0.43 | 0.196 | 1.14 | 0.927 |
|  |  |  |  |  |  |  |  |  |  |
| **Sampling site** | **GIT** (Ref.) | 233 | 13.93 | 1440 | 86.07 | 1 | - | 1 | - |
|  | GUT | 39 | 16.53 | 197 | 83.47 | 1.22 | 0.286 | 3.09 | 0.425 |
|  | others | 38 | 27.34 | 101 | 72.66 | 2.33 | <0.0001 | 1.12 | 0.783 |

**Additional file 1e**. Colistin resistance

|  |  | Resistant isolates | | Susceptible isolates | | One-factorial log. reg. | | Multi-factorial log. reg. | |
| --- | --- | --- | --- | --- | --- | --- | --- | --- | --- |
|  |  | n | % | n | % | OR | p | OR | p |
| **Time period** | **2006-2011** (Ref.) | 167 | 9.63 | 1568 | 90.37 | 1 | - | 1 | - |
|  | 2012-2017 | 159 | 14.87 | 910 | 85.13 | 1.64 | <0.0001 | 1.07 | 0.729 |
|  |  |  |  |  |  |  |  |  |  |
| **Age-group** | **Sow** (Ref.) | 1 | 3.33 | 29 | 96.67 | 1 | - | 1 | - |
|  | Piglet | 4 | 3.45 | 112 | 96.55 | 1.04 | 0.975 | 0.25 | 0.241 |
|  | Nursery | 139 | 21.25 | 515 | 78.75 | 7.83 | 0.044 | 1.86 | 0.575 |
|  | Fattening | 3 | 6.82 | 41 | 93.18 | 2.12 | 0.524 | 0.53 | 0.611 |
|  |  |  |  |  |  |  |  |  |  |
| **Sampling site** | **GIT** (Ref.) | 308 | 13.49 | 1976 | 86.51 | 1 | - | 1 | - |
|  | GUT | 5 | 1.61 | 305 | 98.39 | 0.11 | <0.0001 | <0.0001 | 0.980 |
|  | others | 13 | 6.19 | 197 | 93.81 | 0.42 | 0.003 | 0.96 | 0.931 |

**Additional file 1f**. Enrofloxacin resistance

|  |  | Resistant isolates | | Susceptible isolates | | One-factorial log. reg. | | Multi-factorial log. reg. | |
| --- | --- | --- | --- | --- | --- | --- | --- | --- | --- |
|  |  | n | % | n | % | OR | p | OR | p |
| **Time period** | **2006-2011** (Ref.) | 43 | 2.58 | 1623 | 97.42 | 1 | - | 1 | - |
|  | 2012-2017 | 49 | 4.34 | 1080 | 95.66 | 1.71 | 0.011 | 1.04 | 0.946 |
|  |  |  |  |  |  |  |  |  |  |
| **Age-group** | **Sow** (Ref.) | 6 | 17.14 | 29 | 82.86 | 1 | - | 1 | - |
|  | Piglet | 4 | 3.39 | 114 | 96.61 | 0.17 | 0.009 | 0.19 | 0.118 |
|  | Nursery | 8 | 1.18 | 669 | 98.82 | 0.06 | <0.0001 | 0.1 | 0.029 |
|  | Fattening | 3 | 6.38 | 44 | 93.62 | 0.33 | 0.137 | 0.50 | 0.518 |
|  |  |  |  |  |  |  |  |  |  |
| **Sampling site** | **GIT** (Ref.) | 44 | 1.94 | 2220 | 98.06 | 1 | - | 1 | - |
|  | GUT | 34 | 11 | 275 | 89 | 6.24 | <0.0001 | 1.84 | 0.581 |
|  | others | 14 | 6.31 | 208 | 93.69 | 3.40 | 0.0001 | 2.69 | 0.129 |

**Additional file 1g**. Florfenicol resistance

|  |  | Resistant isolates | | Susceptible isolates | | One-factorial log. reg. | | Multi-factorial log. reg. | |
| --- | --- | --- | --- | --- | --- | --- | --- | --- | --- |
|  |  | n | % | n | % | OR | p | OR | p |
| **Time period** | **2006-2011** (Ref.) | 90 | 7.39 | 1128 | 92.61 | 1 | - | 1 | - |
|  | 2012-2017 | 68 | 8.04 | 778 | 91.96 | 1.10 | 0.586 | 1.89 | 0.139 |
|  |  |  |  |  |  |  |  |  |  |
| **Age-group** | **Sow** (Ref.) | 2 | 8.7 | 21 | 91.3 | 1 | - | 1 | - |
|  | Piglet | 1 | 1.03 | 96 | 98.97 | 0.11 | 0.076 | 0.21 | 0.435 |
|  | Nursery | 34 | 6.64 | 478 | 93.36 | 0.75 | 0.701 | 1.75 | 0.752 |
|  | Fattening | 3 | 9.68 | 28 | 90.32 | 1.13 | 0.902 | 2.37 | 0.625 |
|  |  |  |  |  |  |  |  |  |  |
| **Sampling site** | **GIT** (Ref.) | 121 | 7.09 | 1585 | 92.91 | 1 | - | 1 | - |
|  | GUT | 25 | 14.37 | 149 | 85.63 | 2.20 | 0.0008 | 2.32 | 0.637 |
|  | others | 12 | 6.52 | 172 | 93.48 | 0.91 | 0.774 | 1.56 | 0.455 |

**Additional file 1h**. Gentamicin resistance

|  |  | Resistant isolates | | Susceptible isolates | | One-factorial log. reg. | | Multi-factorial log. reg. | |
| --- | --- | --- | --- | --- | --- | --- | --- | --- | --- |
|  |  | n | % | n | % | OR | p | OR | p |
| **Time period** | **2006-2011** (Ref.) | 66 | 3.73 | 1703 | 96.27 | 1 | - | 1 | - |
|  | 2012-2017 | 68 | 5.51 | 1167 | 94.49 | 1.50 | 0.021 | 0.95 | 0.881 |
|  |  |  |  |  |  |  |  |  |  |
| **Age-group** | **Sow** (Ref.) | 1 | 2.63 | 37 | 97.37 | 1 | - | 1 | - |
|  | Piglet | 5 | 3.57 | 135 | 96.43 | 1.37 | 0.777 | 0.25 | 0.233 |
|  | Nursery | 40 | 5.53 | 683 | 94.47 | 2.17 | 0.451 | 0.36 | 0.366 |
|  | Fattening | 3 | 6.0 | 47 | 94 | 2.36 | 0.465 | 0.42 | 0.485 |
|  |  |  |  |  |  |  |  |  |  |
| **Sampling site** | **GIT** (Ref.) | 112 | 4.61 | 2318 | 95.39 | 1 | - | 1 | - |
|  | GUT | 8 | 2.41 | 324 | 97.59 | 0.51 | 0.070 | <0.001 | 0.978 |
|  | others | 14 | 5.79 | 228 | 94.21 | 1.27 | 0.412 | 0.80 | 0.701 |

**Additional files 1 a-h.** Multifactorial logistic regression analysis with fixed effect time period and factors “age-group” and “sampling site” with respect to apramycin (1a), cefquinome (1b), ceftiofur (1c), cephalothin (1d), colistin (1e), enrofloxacin (1f), florfenicol (1g) and gentamicin (1h). Reference categories for the logistic regression method (time period 2006-2011, sow, gastrointestinal tract) are highlighted in bold and indicated by „Ref.“. One-factorial log. reg.: One-factorial logistic regression model, Multi-factorial log.reg.: Multi-factorial logistic regression model, OR: Point estimate /Odds ratio, p: p-value of the Wald test, n: absolute number of isolates, %: proportion of isolates.
