# Supplementary material for: Phenotypic antimicrobial resistance in Escherichia coli strains isolated from swine husbandries in North Western Germany – temporal patterns in samples from laboratory practice from 2006 to 2017
Source: BMC Vet Res. 2020 Feb 3;16:37. doi: 10.1186/s12917-020-2268-z (PMC6998819; doi:10.1186/s12917-020-2268-z)
Supplement: Supplementary file 2 — Additional file 2. Temporal development in AMR for apramycin (1a), cefquinome (1b), ceftiofur (1c), cephalothin (1d), colistin (1e), enrofloxacin (1f), florfenicol (1 g) and gentamicin (1 h). [file 12917_2020_2268_MOESM2_ESM.doc]

**Additional file 2a.** Temporal development in AMR for apramycin.

|  | **number of isolates with MIC values (mg/ml) of…** | | | | **s r** | | | | | | |
| --- | --- | --- | --- | --- | --- | --- | --- | --- | --- | --- | --- |
|  | **4** | **16** | **32** | **64** | **n** | **MIC _50_** | **MIC _90_** | **n** | **%** | **n** | **%** |
| **year** |  |  |  |  |  |  |  |  |  |  |  |
| **2006** | 291 | 2 | 1 | 16 | 310 | 4.00 | 4.00 | 293 | 94.52 | 17 | 5.48 |
| **2007** | 359 | 2 | 2 | 18 | 381 | 4.00 | 4.00 | 361 | 94.75 | 20 | 5.25 |
| **2008** | 292 | 8 | . | 9 | 309 | 4.00 | 4.00 | 300 | 97.09 | 9 | 2.91 |
| **2009** | 249 | 10 | 3 | 8 | 270 | 4.00 | 4.00 | 259 | 95.93 | 11 | 4.07 |
| **2010** | 248 | 5 | 6 | 11 | 270 | 4.00 | 4.00 | 253 | 93.70 | 17 | 6.30 |
| **2011** | 250 | 4 | . | 12 | 266 | 4.00 | 4.00 | 254 | 95.49 | 12 | 4.51 |
| **2012** | 242 | 4 | 1 | 14 | 261 | 4.00 | 4.00 | 246 | 94.25 | 15 | 5.75 |
| **2013** | 271 | 10 | . | 15 | 296 | 4.00 | 4.00 | 281 | 94.93 | 15 | 5.07 |
| **2014** | 194 | 3 | 2 | 7 | 206 | 4.00 | 4.00 | 197 | 95.63 | 9 | 4.37 |
| **2015** | 183 | 4 | . | 7 | 194 | 4.00 | 4.00 | 187 | 96.39 | 7 | 3.61 |
| **2016** | 156 | 1 | . | 10 | 167 | 4.00 | 4.00 | 157 | 94.01 | 10 | 5.99 |
| **2017** | 34 | . | . | 5 | 39 | 4.00 | 64.00 | 34 | 87.18 | 5 | 12.82 |
| **total** | 2769 | 53 | 15 | 132 | 2969 | 4.00 | 4.00 | 2822 | 95.05 | 147 | 4.95 |

**Additional file 2b.** Temporal development in AMR for cefquinome.

|  | **number of isolates with MIC values (mg/ml) of…** | | | | | **s i** | | | | | | | **r** | |
| --- | --- | --- | --- | --- | --- | --- | --- | --- | --- | --- | --- | --- | --- | --- |
|  | **0.5** | **2** | **4** | **8** | **16** | **n** | **MIC _50_** | **MIC _90_** | **n** | **%** | **n** | **%** | **n** | **%** |
| **year** |  |  |  |  |  |  |  |  |  |  |  |  |  |  |
| **2006** | 304 | . | 3 | . | 3 | 310 | 0.50 | 0.50 | 304 | 98.06 | 3 | 0.97 | 3 | 0.97 |
| **2007** | 373 | 1 | 1 | 1 | 5 | 381 | 0.50 | 0.50 | 374 | 98.16 | 1 | 0.26 | 6 | 1.57 |
| **2008** | 300 | 1 | 1 | 3 | 4 | 309 | 0.50 | 0.50 | 301 | 97.41 | 1 | 0.32 | 7 | 2.27 |
| **2009** | 254 | 3 | . | . | 13 | 270 | 0.50 | 0.50 | 257 | 95.19 | . | . | 13 | 4.81 |
| **2010** | 262 | . | . | 3 | 5 | 270 | 0.50 | 0.50 | 262 | 97.04 | . | . | 8 | 2.96 |
| **2011** | 251 | 2 | 3 | 4 | 6 | 266 | 0.50 | 0.50 | 253 | 95.11 | 3 | 1.13 | 10 | 3.76 |
| **2012** | 248 | . | 1 | 3 | 9 | 261 | 0.50 | 0.50 | 248 | 95.02 | 1 | 0.38 | 12 | 4.60 |
| **2013** | 276 | 1 | 2 | 4 | 13 | 296 | 0.50 | 0.50 | 277 | 93.58 | 2 | 0.68 | 17 | 5.74 |
| **2014** | 197 | . | . | 1 | 8 | 206 | 0.50 | 0.50 | 197 | 95.63 | . | . | 9 | 4.37 |
| **2015** | 185 | 2 | 1 | 1 | 5 | 194 | 0.50 | 0.50 | 187 | 96.39 | 1 | 0.52 | 6 | 3.09 |
| **2016** | 164 | . | . | . | 3 | 167 | 0.50 | 0.50 | 164 | 98.20 | . | . | 3 | 1.80 |
| **2017** | 37 | . | . | . | 2 | 39 | 0.50 | 0.50 | 37 | 94.87 | . | . | 2 | 5.13 |
| **total** | 2851 | 10 | 12 | 20 | 76 | 2969 | 0.50 | 0.50 | 2861 | 96.36 | 12 | 0.40 | 96 | 3.23 |

**Additional file 2c.** Temporal development in AMR for ceftiofur.

|  | **number of isolates with MIC values (mg/ml) of…** | | | | | | | **s i** | | | | | | | | **r** | |
| --- | --- | --- | --- | --- | --- | --- | --- | --- | --- | --- | --- | --- | --- | --- | --- | --- | --- |
|  | **0.063** | **0.25** | **0.5** | **2** | **4** | **8** | **16** | **n** | **MIC _50_** | **MIC _90_** | **n** | **%** | **n** | **%** | **n** | | **%** |
| **year** |  |  |  |  |  |  |  |  |  |  |  |  |  |  |  | |  |
| **2006** | . | . | 302 | 2 | . | . | 6 | 310 | 0.50 | 0.50 | 304 | 98.06 | . | . | 6 | | 1.94 |
| **2007** | . | . | 371 | 1 | 3 | . | 6 | 381 | 0.50 | 0.50 | 372 | 97.64 | 3 | 0.79 | 6 | | 1.57 |
| **2008** | . | . | 299 | . | 1 | 2 | 7 | 309 | 0.50 | 0.50 | 299 | 96.76 | 1 | 0.32 | 9 | | 2.91 |
| **2009** | . | . | 254 | 3 | . | . | 13 | 270 | 0.50 | 0.50 | 257 | 95.19 | . | . | 13 | | 4.81 |
| **2010** | . | . | 260 | 1 | 1 | . | 8 | 270 | 0.50 | 0.50 | 261 | 96.67 | 1 | 0.37 | 8 | | 2.96 |
| **2011** | . | . | 251 | 1 | 2 | 2 | 10 | 266 | 0.50 | 0.50 | 252 | 94.74 | 2 | 0.75 | 12 | | 4.51 |
| **2012** | . | . | 247 | . | . | 2 | 12 | 261 | 0.50 | 0.50 | 247 | 94.64 | . | . | 14 | | 5.36 |
| **2013** | . | . | 274 | 3 | . | 1 | 18 | 296 | 0.50 | 0.50 | 277 | 93.58 | . | . | 19 | | 6.42 |
| **2014** | . | . | 196 | . | 1 | . | 9 | 206 | 0.50 | 0.50 | 196 | 95.15 | 1 | 0.49 | 9 | | 4.37 |
| **2015** | . | . | 187 | . | . | . | 7 | 194 | 0.50 | 0.50 | 187 | 96.39 | . | . | 7 | | 3.61 |
| **2016** | . | . | 161 | 2 | . | . | 4 | 167 | 0.50 | 0.50 | 163 | 97.60 | . | . | 4 | | 2.40 |
| **2017** | 1 | 62 | 56 | . | . | . | 5 | 124 | 0.25 | 0.50 | 119 | 95.97 | . | . | 5 | | 4.03 |
| **total** | 1 | 62 | 2858 | 13 | 8 | 7 | 105 | 3054 | 0.50 | 0.50 | 2934 | 96.07 | 8 | 0.26 | 112 | | 3.67 |

**Additional file 2d.** Temporal development in AMR for cephalothin.

|  | **number of isolates with MIC values (mg/ml) of…** | | | | | |  | **s i** | | | | | | **r** | |
| --- | --- | --- | --- | --- | --- | --- | --- | --- | --- | --- | --- | --- | --- | --- | --- |
|  | **2** | **4** | **8** | **16** | **32** | **64** | **n** | **MIC _50_** | **MIC _90_** | **n** | **%** | **n** | **%** | **n** | **%** |
| **year** |  |  |  |  |  |  |  |  |  |  |  |  |  |  |  |
| **2006** | 52 | . | 138 | 94 | 18 | 8 | 310 | 8.00 | 16.00 | 190 | 61.29 | 94 | 30.32 | 26 | 8.39 |
| **2007** | 37 | . | 180 | 124 | 21 | 19 | 381 | 8.00 | 32.00 | 217 | 56.96 | 124 | 32.55 | 40 | 10.50 |
| **2008** | 33 | . | 148 | 101 | 14 | 13 | 309 | 8.00 | 16.00 | 181 | 58.58 | 101 | 32.69 | 27 | 8.74 |
| **2009** | 15 | . | 122 | 97 | 13 | 23 | 270 | 8.00 | 32.00 | 137 | 50.74 | 97 | 35.93 | 36 | 13.33 |
| **2010** | 30 | . | 125 | 91 | 11 | 13 | 270 | 8.00 | 16.00 | 155 | 57.41 | 91 | 33.70 | 24 | 8.89 |
| **2011** | 55 | . | 142 | 50 | 3 | 16 | 266 | 8.00 | 16.00 | 197 | 74.06 | 50 | 18.80 | 19 | 7.14 |
| **2012** | 27 | . | 129 | 80 | 10 | 15 | 261 | 8.00 | 16.00 | 156 | 59.77 | 80 | 30.65 | 25 | 9.58 |
| **2013** | 12 | . | 114 | 136 | 12 | 22 | 296 | 16.00 | 32.00 | 126 | 42.57 | 136 | 45.95 | 34 | 11.49 |
| **2014** | 20 | . | 104 | 63 | 9 | 10 | 206 | 8.00 | 16.00 | 124 | 60.19 | 63 | 30.58 | 19 | 9.22 |
| **2015** | 18 | . | 76 | 80 | 11 | 9 | 194 | 16.00 | 32.00 | 94 | 48.45 | 80 | 41.24 | 20 | 10.31 |
| **2016** | 10 | . | 82 | 55 | 15 | 5 | 167 | 8.00 | 32.00 | 92 | 55.09 | 55 | 32.93 | 20 | 11.98 |
| **2017** | 2 | 6 | 61 | 35 | 17 | 3 | 124 | 8.00 | 32.00 | 69 | 55.65 | 35 | 28.23 | 20 | 16.13 |
| **total** | 311 | 6 | 1421 | 1006 | 154 | 156 | 3054 | 8.00 | 32.00 | 1738 | 56.91 | 1006 | 32.94 | 310 | 10.15 |

**Additional file 2e.** Temporal development in AMR for colistin.

|  | **number of isolates with MIC values (mg/ml) of…** | | | | | **s i** | | | | | | | **r** | | |
| --- | --- | --- | --- | --- | --- | --- | --- | --- | --- | --- | --- | --- | --- | --- | --- |
|  | **0.25** | **1** | **2** | **4** | **8** | **n** | **MIC _50_** | **MIC _90_** | **n** | **%** | **n** | **%** | | **n** | **%** |
| **year** |  |  |  |  |  |  |  |  |  |  |  |  | |  |  |
| **2006** | 276 | 9 | 9 | 7 | 9 | 310 | 0.25 | 1.00 | 276 | 89.03 | 18 | 5.81 | | 16 | 5.16 |
| **2007** | 335 | 11 | 7 | 10 | 18 | 381 | 0.25 | 1.00 | 335 | 87.93 | 18 | 4.72 | | 28 | 7.35 |
| **2008** | 274 | 4 | 3 | 8 | 20 | 309 | 0.25 | 2.00 | 274 | 88.67 | 7 | 2.27 | | 28 | 9.06 |
| **2009** | 233 | 12 | . | 11 | 14 | 270 | 0.25 | 1.00 | 233 | 86.30 | 12 | 4.44 | | 25 | 9.26 |
| **2010** | 228 | 4 | 4 | 7 | 27 | 270 | 0.25 | 6.00 | 228 | 84.44 | 8 | 2.96 | | 34 | 12.59 |
| **2011** | 222 | 5 | 3 | 11 | 25 | 266 | 0.25 | 4.00 | 222 | 83.46 | 8 | 3.01 | | 36 | 13.53 |
| **2012** | 174 | 50 | 1 | 4 | 32 | 261 | 0.25 | 8.00 | 174 | 66.67 | 51 | 19.54 | | 36 | 13.79 |
| **2013** | 234 | 27 | 2 | 7 | 26 | 296 | 0.25 | 4.00 | 234 | 79.05 | 29 | 9.80 | | 33 | 11.15 |
| **2014** | 161 | 18 | 1 | 7 | 19 | 206 | 0.25 | 4.00 | 161 | 78.16 | 19 | 9.22 | | 26 | 12.62 |
| **2015** | 88 | 73 | 2 | 4 | 27 | 194 | 1.00 | 8.00 | 88 | 45.36 | 75 | 38.66 | | 31 | 15.98 |
| **2016** | 143 | 2 | 1 | 11 | 9 | 166 | 0.25 | 4.00 | 143 | 86.14 | 3 | 1.81 | | 20 | 12.05 |
| **2017** | 110 | 1 | . | 10 | 3 | 124 | 0.25 | 4.00 | 110 | 88.71 | 1 | 0.81 | | 13 | 10.48 |
| **total** | 2478 | 216 | 33 | 97 | 229 | 3053 | 0.25 | 4.00 | 2478 | 81.17 | 249 | 8.16 | | 326 | 10.68 |

**Additional file 2f.** Temporal development in AMR for enrofloxacin.

|  | **number of isolates with MIC values (mg/ml) of…** | | | | | | | | | **s i** | | | | | | | **r** | | |
| --- | --- | --- | --- | --- | --- | --- | --- | --- | --- | --- | --- | --- | --- | --- | --- | --- | --- | --- | --- |
|  | **0.016** | **0.031** | **0.063** | **0.125** | **0.25** | **0.5** | **1** | **2** | **4** | **n** | **MIC _50_** | **MIC _90_** | **n** | **%** | **n** | **%** | | **n** | **%** |
| **year** |  |  |  |  |  |  |  |  |  |  |  |  |  |  |  |  | |  |  |
| **2006** | . | 253 | . | 10 | 23 | 14 | 2 | . | 8 | 310 | 0.03 | 0.25 | 286 | 92.26 | 16 | 5.16 | | 8 | 2.58 |
| **2007** | . | 322 | . | 3 | 20 | 26 | 2 | 1 | 7 | 381 | 0.03 | 0.25 | 345 | 90.55 | 28 | 7.35 | | 8 | 2.10 |
| **2008** | . | 251 | . | 4 | 19 | 24 | 5 | 1 | 5 | 309 | 0.03 | 0.50 | 274 | 88.67 | 29 | 9.39 | | 6 | 1.94 |
| **2009** | . | 226 | . | 6 | 10 | 19 | 5 | 1 | 3 | 270 | 0.03 | 0.50 | 242 | 89.63 | 24 | 8.89 | | 4 | 1.48 |
| **2010** | . | 210 | . | 10 | 17 | 25 | 2 | 2 | 4 | 270 | 0.03 | 0.50 | 237 | 87.78 | 27 | 10.00 | | 6 | 2.22 |
| **2011** | . | 216 | . | 7 | 16 | 13 | 3 | 1 | 10 | 266 | 0.03 | 0.50 | 239 | 89.85 | 16 | 6.02 | | 11 | 4.14 |
| **2012** | . | 213 | . | 4 | 15 | 20 | 2 | 2 | 5 | 261 | 0.03 | 0.50 | 232 | 88.89 | 22 | 8.43 | | 7 | 2.68 |
| **2013** | . | 237 | . | 4 | 12 | 23 | 10 | 3 | 7 | 296 | 0.03 | 0.50 | 253 | 85.47 | 33 | 11.15 | | 10 | 3.38 |
| **2014** | . | 158 | . | 6 | 11 | 22 | 3 | 1 | 5 | 206 | 0.03 | 0.50 | 175 | 84.95 | 25 | 12.14 | | 6 | 2.91 |
| **2015** | . | 153 | . | 1 | 9 | 16 | 1 | 4 | 10 | 194 | 0.03 | 0.50 | 163 | 84.02 | 17 | 8.76 | | 14 | 7.22 |
| **2016** | . | 130 | . | 4 | 12 | 10 | 2 | 2 | 7 | 167 | 0.03 | 0.50 | 146 | 87.43 | 12 | 7.19 | | 9 | 5.39 |
| **2017** | 63 | 33 | 5 | 3 | 7 | 9 | 1 | 2 | 1 | 124 | 0.02 | 0.50 | 111 | 89.52 | 10 | 8.06 | | 3 | 2.42 |
| **total** | 63 | 2402 | 5 | 62 | 171 | 221 | 38 | 20 | 72 | 3054 | 0.03 | 0.50 | 2703 | 88.51 | 259 | 8.48 | | 92 | 3.01 |

**Additional file 2g.** Temporal development in AMR for florfenicol.

|  | **number of isolates with MIC values (mg/ml) of…** | | | | | **s i** | | | | | | | **r** | | |
| --- | --- | --- | --- | --- | --- | --- | --- | --- | --- | --- | --- | --- | --- | --- | --- |
|  | **0.5** | **2** | **4** | **8** | **16** | **n** | **MIC _50_** | **MIC _90_** | **n** | **%** | **n** | **%** | | **n** | **%** |
| **year** |  |  |  |  |  |  |  |  |  |  |  |  | |  |  |
| **2006** | 5 | 40 | 177 | 77 | 11 | 310 | 4.00 | 8.00 | 222 | 71.61 | 77 | 24.84 | | 11 | 3.55 |
| **2007** | 6 | 31 | 198 | 119 | 27 | 381 | 4.00 | 8.00 | 235 | 61.68 | 119 | 31.23 | | 27 | 7.09 |
| **2008** | 2 | 27 | 149 | 120 | 11 | 309 | 4.00 | 8.00 | 178 | 57.61 | 120 | 38.83 | | 11 | 3.56 |
| **2009** | 4 | 20 | 144 | 88 | 14 | 270 | 4.00 | 8.00 | 168 | 62.22 | 88 | 32.59 | | 14 | 5.19 |
| **2010** | . | 13 | 141 | 101 | 15 | 270 | 4.00 | 8.00 | 154 | 57.04 | 101 | 37.41 | | 15 | 5.56 |
| **2011** | 1 | 22 | 148 | 83 | 12 | 266 | 4.00 | 8.00 | 171 | 64.29 | 83 | 31.20 | | 12 | 4.51 |
| **2012** | 1 | 6 | 146 | 94 | 14 | 261 | 4.00 | 8.00 | 153 | 58.62 | 94 | 36.02 | | 14 | 5.36 |
| **2013** | 2 | 8 | 155 | 108 | 23 | 296 | 4.00 | 8.00 | 165 | 55.74 | 108 | 36.49 | | 23 | 7.77 |
| **2014** | 1 | 5 | 117 | 76 | 7 | 206 | 4.00 | 8.00 | 123 | 59.71 | 76 | 36.89 | | 7 | 3.40 |
| **2015** | . | 17 | 127 | 38 | 12 | 194 | 4.00 | 8.00 | 144 | 74.23 | 38 | 19.59 | | 12 | 6.19 |
| **2016** | 1 | 14 | 92 | 55 | 5 | 167 | 4.00 | 8.00 | 107 | 64.07 | 55 | 32.93 | | 5 | 2.99 |
| **2017** | . | 15 | 71 | 31 | 7 | 124 | 4.00 | 8.00 | 86 | 69.35 | 31 | 25.00 | | 7 | 5.65 |
| **total** | 23 | 218 | 1665 | 990 | 158 | 3054 | 4.00 | 8.00 | 1906 | 62.41 | 990 | 32.42 | | 158 | 5.17 |

**Additional file 2h.** Temporal development in AMR for gentamicin.

|  | **number of isolates with MIC values (mg/ml) of…** | | | | | | | **s i** | | | | | | | | **r** | |
| --- | --- | --- | --- | --- | --- | --- | --- | --- | --- | --- | --- | --- | --- | --- | --- | --- | --- |
|  | **0.125** | **0.5** | **1** | **4** | **8** | **16** | **32** | **n** | **MIC 50** | **MIC 90** | **n** | **%** | **n** | **%** | **n** | | **%** |
| **year** |  |  |  |  |  |  |  |  |  |  |  |  |  |  |  | |  |
| **2006** | . | . | 291 | 6 | 9 | 1 | 3 | 310 | 1.00 | 1.00 | 297 | 95.81 | 9 | 2.90 | 4 | | 1.29 |
| **2007** | . | . | 355 | 7 | 6 | 8 | 5 | 381 | 1.00 | 1.00 | 362 | 95.01 | 6 | 1.57 | 13 | | 3.41 |
| **2008** | . | . | 290 | 3 | 5 | 6 | 5 | 309 | 1.00 | 1.00 | 293 | 94.82 | 5 | 1.62 | 11 | | 3.56 |
| **2009** | . | . | 250 | 4 | 5 | 9 | 2 | 270 | 1.00 | 1.00 | 254 | 94.07 | 5 | 1.85 | 11 | | 4.07 |
| **2010** | . | . | 238 | 10 | 9 | 5 | 8 | 270 | 1.00 | 4.00 | 248 | 91.85 | 9 | 3.33 | 13 | | 4.81 |
| **2011** | . | . | 245 | 4 | 3 | 4 | 10 | 266 | 1.00 | 1.00 | 249 | 93.61 | 3 | 1.13 | 14 | | 5.26 |
| **2012** | . | . | 243 | 1 | 3 | 9 | 5 | 261 | 1.00 | 1.00 | 244 | 93.49 | 3 | 1.15 | 14 | | 5.36 |
| **2013** | . | . | 269 | 6 | 4 | 8 | 9 | 296 | 1.00 | 1.00 | 275 | 92.91 | 4 | 1.35 | 17 | | 5.74 |
| **2014** | . | . | 193 | 4 | . | 5 | 4 | 206 | 1.00 | 1.00 | 197 | 95.63 | . | . | 9 | | 4.37 |
| **2015** | . | . | 182 | 2 | 1 | 3 | 6 | 194 | 1.00 | 1.00 | 184 | 94.85 | 1 | 0.52 | 9 | | 4.64 |
| **2016** | . | . | 151 | 2 | 2 | 7 | 5 | 167 | 1.00 | 1.00 | 153 | 91.62 | 2 | 1.20 | 12 | | 7.19 |
| **2017** | 10 | 52 | 50 | 2 | 3 | 6 | 1 | 124 | 0.75 | 1.00 | 114 | 91.94 | 3 | 2.42 | 7 | | 5.65 |
| **total** | 10 | 52 | 2757 | 51 | 50 | 71 | 63 | 3054 | 1.00 | 1.00 | 2870 | 93.98 | 50 | 1.64 | 134 | | 4.39 |
